# Hydrostable hard tissue adhesives based on organophosphates and magnesium phosphates with strong bonding and cellular compatibility

**DOI:** 10.1038/s41598-025-15174-7

**Published:** 2025-08-14

**Authors:** Tobias Renner, Valentin Carl Steinacker, Niclas Fleckenstein, Laura Marx, Alexander Kübler, Paul Frederik Otto, Uwe Gbureck

**Affiliations:** 1 Department of Oral and Maxillofacial Plastic Surgery, University Hospital Würzburg, Pleicherwall 2, 97070 Würzburg, Germany; 2 Department for Functional Materials in Medicine and Dentistry, University Hospital Würzburg, Pleicherwall 2, 97070 Würzburg, Germany

**Keywords:** Biomaterials - cells, Biomedical materials, Biomineralization, Tissues, Ceramics, Mechanical properties, Bioinspired materials, Biomedical engineering, Bioinspired materials, Trauma, Fracture repair

## Abstract

Bone adhesives have significant potential for improving surgical procedures and can enhance and simplify them. Recently, phosphoserine-modified mineral-organic resorbable bone adhesives have shown particular promise. Among them, MgO/MgP-based cement exhibit high adhesive strength but suffer from water instability. This study introduces mineral-organic hard tissue adhesives based on organophosphates and magnesium phosphate with hydrolytic stability to enable cell testing. Amorphised magnesium phosphates enhanced reactivity, eliminating MgO additives. Alongside the established organophosphate phosphoserine (OPLS), sodium phytate was investigated as an alternative. We performed comprehensive mechanical and material characterizations, complemented by in vitro cytocompatibility assessments using osteoblastic MG-63 cells and osteoclastic RAW 264.7 cells differentiated with RANKL. Sodium phytate demonstrated strong adhesive properties, with an initial shear strength of 4.35 ± 0.86 MPa, retaining 1.3 ± 0.27 MPa after 7 days. However, it showed lower cytocompatibility with MG-63 cells than with OPLS. The OPLS/MgP combination exhibited both favorable cytocompatibility and strong adhesive performance, achieving an initial shear strength of 3.87 ± 0.53 MPa and maintaining 2.19 ± 0.69 MPa after 7 days. These findings underscore the potential of organophosphate-magnesium compound cement as viable candidates for mineral-organic hard tissue adhesives in surgical applications, combining excellent adhesive properties with favorable cytocompatibility.

## Introduction

Bone adhesives are of particular relevance in situations where conventional fixation methods are limited, such as comminuted fractures or thin bone structures in regions like the facial skeleton or maxillary sinus. In addition, they enable novel therapeutic approaches by facilitating the temporary fixation of plates or bone fragments in anatomically challenging areas, the stabilization of implants, or the adhesion of osteosynthesis meshes and membranes, to name but a few. Cementitious formulations may further contribute by combining adhesive fixation with the capacity to fill bone defects and support regeneration. Despite decades of research, no bone adhesive has gained widespread acceptance due to the considerable challenges they face, such as hardening under adverse conditions and maintaining biocompatibility. Traditional bone cements, while biocompatible, lack adhesive properties and are susceptible to moisture, limiting their utility as bone adhesives. Recent advancements in bone adhesive research, particularly in mineral-organic resorbable bone adhesives (MORBA), show promise^1–6^. MORBA, composed of phosphoserine and calcium compounds, represent a departure from conventional cements, utilizing components familiar in classical bone cement formulations. Notably, phosphoserine emerges as a primary reactant in MORBA formulations, distinguishing them from traditional bone cements. Although the subject of bone adhesives in general is still a marginal topic, the term phosphoserine-modified cements (PMC) is already becoming established in specialist contexts, which underlines the increasing importance of phosphoserine in the field of medical adhesives. Spicer et al.^7^ see the carboxyl and phosphoryl groups in particular as key functional groups for adhesion^7^. Their studies are not limited to phosphoserine, but also examine phosphothreonine or phosphotyrosine. The phosphorylated amino acids mentioned above are generally assigned to organophosphates. Phytic acid is a molecule that is occasionally used in bone replacement materials and can be classified as an organophosphate. As a chelating agent, it can ensure improved regulation of curing^8^. Cement with phytic acid as an additive also show comparably better bioactivity and improve mechanical properties^8,9^. However, its use in the context of adhesive bone replacement materials or bone adhesives is only occasionally found^10,11^. The salt of phytic acid (IP6), sodium phytate (Na-IP6), has played a comparatively minor role in the context of bone regeneration to date. However, there are indications that it can act as a potent crosslinker of bioactive glasses, which may be superior to conventional crosslinkers in terms of biocompatibility^12^.

While calcium phosphates remain prominent in bone replacement materials^13^, recent studies highlight the significance of magnesium in bone regeneration mechanisms^14^ and the superiority that magnesium phosphate cements may have over calcium phosphate cements^15,16^. Biomineral adhesive bone cements incorporating magnesium oxides or phosphates, structurally categorized as MORBA, have demonstrated potential for superior adhesive properties^17^. However, particularly high-performance adhesives based on MgO, farringtonite and phosphoserine are not stable in water in the long term^17^. Less performant adhesives based on phytic acid, MgO and farringtonite, on the other hand, are stable in water^10^. The phytic acid-based water-stable bone adhesives by Brückner et al. (2019)^10^ outreaches the minimum adhesive strength of > 0.2 MPa postulated by Weber and Chapman^18^. However, with values of > 0.8 MPa after seven days of storage^10^, further optimization appears necessary. Phytate ions are suggested to influence adhesion^10^, whereas phosphoserine, offering greater flexibility due to its additional amino group, has yet been utilized in some adhesive experiments as described above. Such formulations require more reactive Mg^2^⁺ sources, such as higher amounts of MgO, which go in solution^17^. However, increased MgO concentrations are associated with increased hydrolysis susceptibility^17^. Despite the mentioned limitations, this new class of magnesium-based adhesive cements is highly promising due to its strong affinity for organophosphates. This innovation is not limited to use as a bone adhesive, but should rather be seen as a platform from which many medical working materials can emerge. It has considerable potential for developing new treatment methods with far-reaching medical applications.

This work developed bone adhesives based on organophosphates and magnesium compounds. By using sodium phytate instead of phytic acid, higher organophosphate contents were achieved, enhancing adhesiveness significantly. Instead of the commonly used MgO and farringtonite, more soluble magnesium phosphate hydrates were utilized to provide reactive ions, accelerating the reaction with sodium phytate and supporting a uniform cement matrix. Additionally, phosphoserine was tested as an alternative organophosphate, offering greater flexibility and improved surface adhesion due to its molecular structure. A key focus was the hydrolytic stability of the adhesives, essential not only for clinical applications but also for in-vitro cytocompatibility tests. Ultimately, this study emphasizes the biological performance of these innovative materials.

## Results

A significant part of this work was dedicated to defining the precise formulations presented in Table 1. To achieve this, numerous combinations of potential formulations were systematically assessed, and various ratios of powders were mixed with water. Different powder-to-liquid ratios (PLR) were also explored to determine the optimal parameters. The key point was the heat treatment of the TMP hydrates, which allowed a sufficient reaction rate to be achieved.

**Table 1:** Labelling and makeup of the designed compositions (PLR = powder-to-liquid ratio).

Labelling	Composition		PLR
(1)	Na-IP6	330 mg	3.53
	Commercial TMP hydrate (Mg_3_(PO_4_)_2_·xH_2_O, heat-treated)	200 mg	
	H_2_O	150 µl	
(2)	Na-IP6	510 mg	2.96
	Cattiite (Mg_3_(PO_4_)_2_·22H_2_O, heat-treated)	200 mg	
	H_2_O	240 µl	
(3)	OPLS	160 mg	2.33
	Commercial TMP hydrate (Mg_3_(PO_4_)_2_·xH_2_O, heat-treated)	400 mg	
	H_2_O	240 µl	
(4)	OPLS	240 mg	2.13
	Cattiite (Mg_3_(PO_4_)_2_·22H_2_O, heat-treated)	400 mg	
	H_2_O	300 µl	
(5) (reference)	IP6 (25%)	150 µl	2.01
	MgO (2933)	23 mg	
	Farringtonite (Mg_3_(PO_4_)_2_)	278 mg	
(6) (reference)	OPLS	150 mg	4.23
	TTCP (Ca_4_(PO_4_)_2_O)	400 mg	
	H_2_O	130 mg	

**Fig. 1:**
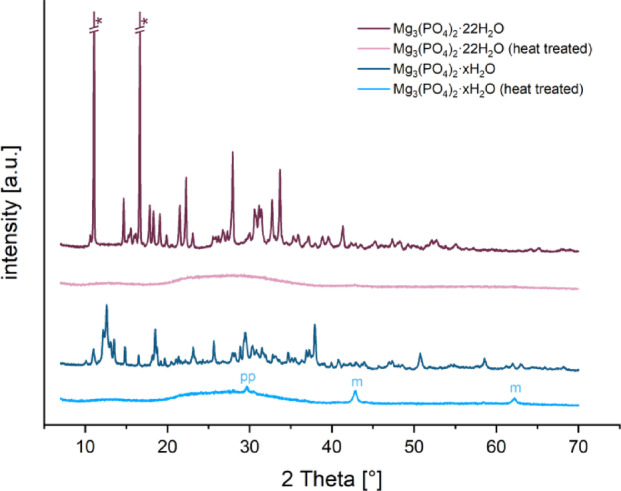
Diffraction patterns of Mg_3_(PO_4_)_2_·22H_2_O (purple) and Mg_3_(PO_4_)_2_·xH_2_O (blue) before (dark color) and after (light color) heat treatment.

Figure 1 shows XRD patterns of cattiite and TMP hydrate before and after heat treatment. Cattiite is phase-pure and becomes amorphous after heating, with a residual peak at 2θ ≈ 42° for magnesium pyrophosphate. TMP hydrate shows mixed phases (newberyite, magnesium phosphate octa- and decahydrates, magnesium hydroxide) and retains peaks for magnesium pyrophosphate and magnesium oxide after heating.

**Fig. 2:**
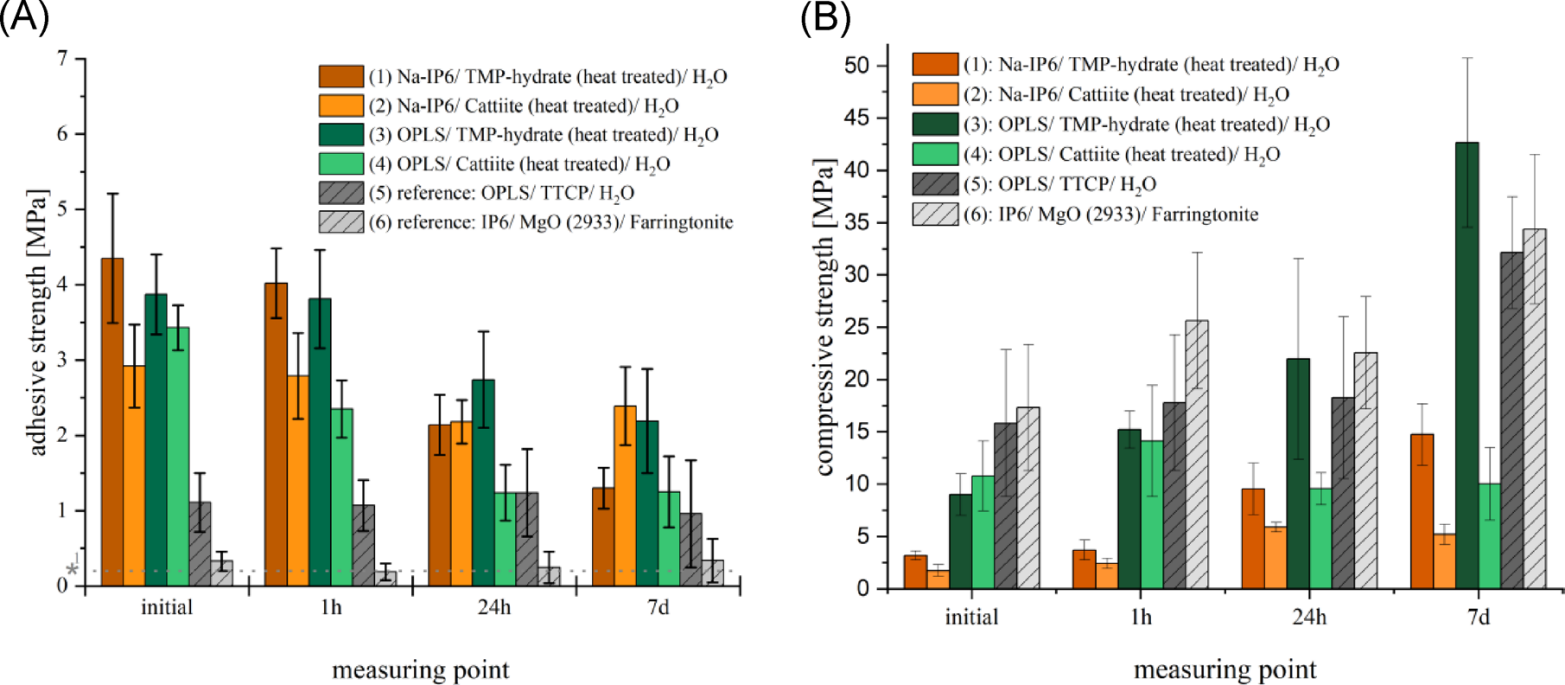
Adhesive strength (**A**) of cement cuboids and Compressive strength (**B**) on bovine cortical bone substrates of cements made from: brown: C_6_H_6_Na_12_O_24_P_6_/ Mg_3_(PO_4_)_2_·xH_2_O (heat treated)/ H_2_O, orange: C_6_H_6_Na_12_O_24_P_6_/ Mg_3_(PO_4_)_2_·22H_2_O (heat treated)/ H_2_O , dark green: OPLS/ Mg_3_(PO_4_)_2_·xH_2_O (heat treated)/ H_2_O, light green: OPLS/ Mg_3_(PO_4_)_2_·22H_2_O (heat treated)/ H_2_O, dark gray: OPLS/ TTCP/ H_2_O (reference), light gray: C_6_H_18_O_24_P_6_/ MgO (2933)/ Mg_3_(PO_4_)_2_. Measurements occurred initially or after 1 h, 24 h, or 7 days of hardening at 37 °C (n = 10, respectively).*1: Minimum adhesive strength requirement postulated by Weber and Chapman in order to be suitable as a bone adhesive^18^.

Adhesive strength and compressive strength values are illustrated in Fig. 2. Compositions (1) and (2) exhibited low initial compressive strengths, which increased over seven days (composition (1): 3.19–14.76 MPa; composition (2): 1.75–5.2 MPa). O-phospho-L-serine (OPLS)-based composition (3) showed high values, reaching up to 42.66 MPa. Adhesive strengths varied, with composition (1) peaking initially (4.35 MPa) but declining over time, and composition (2) maintaining the highest strength after seven days (2.39 MPa). Reference materials consistently displayed significantly lower strengths, ranging between approx. 0.2 to 1.2 MPa.

A two-way ANOVA showed significant effects of cement composition, storage time, and their interaction on shear strength (*p* < 0.001). Tukey’s post-hoc test revealed that all four experimental formulations (1)–(4) had significantly higher initial shear strength than both references (*p* < 0.01). Reference (5) outperformed reference (6) initially (1.11 ± 0.39 vs. 0.33 ± 0.13 MPa), though this difference narrowly missed significance (*p* = 0.099). After 7 days, all experimental formulations remained significantly stronger than reference (6) (*p* < 0.01), while reference (5) no longer differed significantly from reference (6) or from lower-performing experimental groups like formulation (4).

Compressive strength also differed significantly by formulation, time point, and their interaction (two-way ANOVA, *p* < 0.0001 for all). Experimental formulations containing sodium phytate (Na-IP6) showed significantly lower values than the other groups at both time points (*p* < 0.0001 at day 7). Although formulation (3) exceeded both references at day 7, the differences were not statistically significant (*p* > 0.05).

**Fig. 3:**
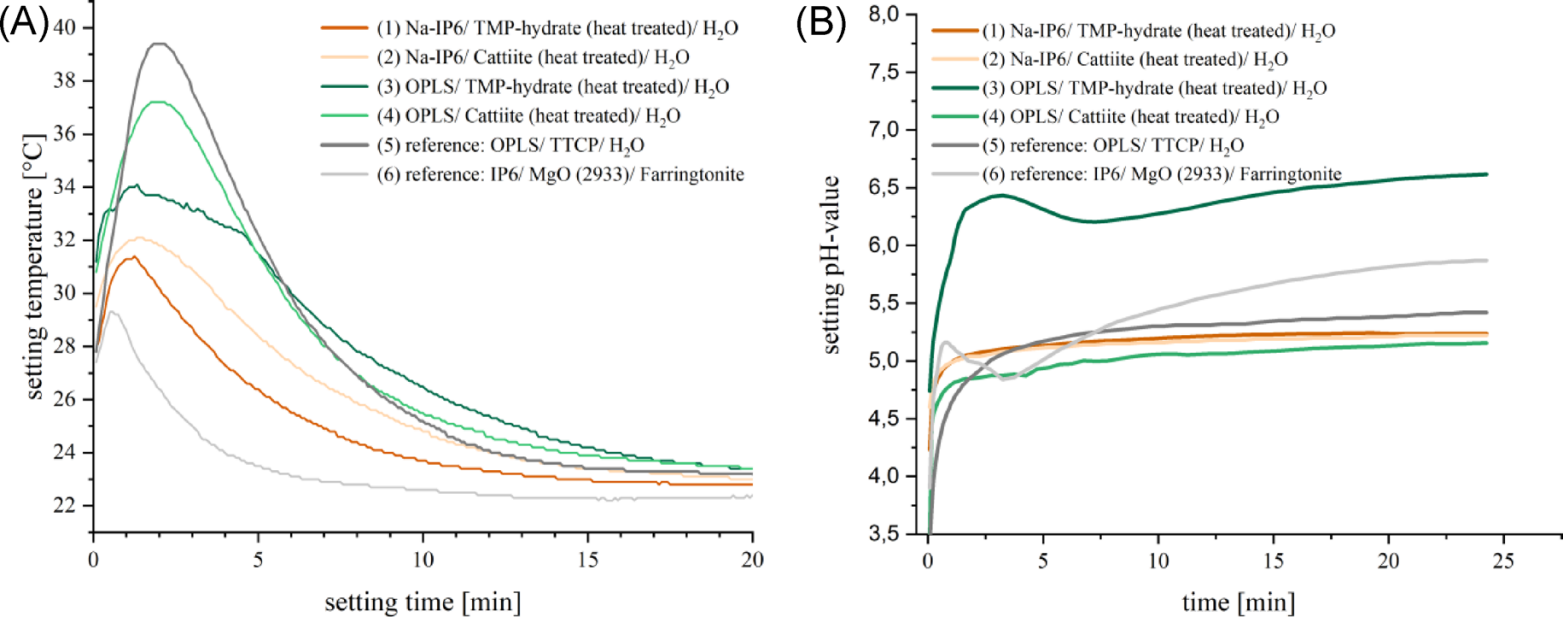
Temperature (**A**) and pH (**B**) development during the setting reaction of the tested adhesive cements.

pH value and temperature development during the setting reaction are shown in Fig. 3. The maximum temperature is reached within 3 min for all cements, with none exceeding 40 °C. pH values ranged from 5.1 to 6.7, with composition (3) being slightly more neutral than the reference cement. Unlike the others, compositions (3) and (6) show a biphasic saturation curve.

**Fig. 4:**
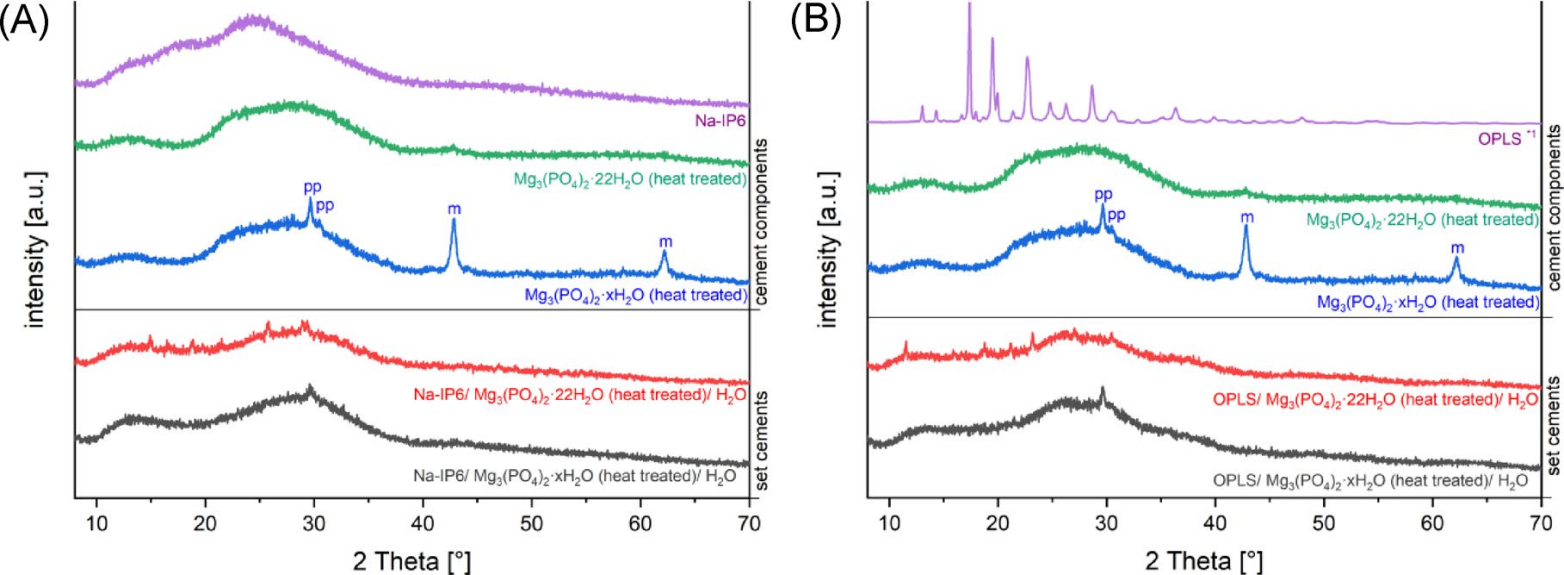
Diffraction patterns of (**A**): Na-IP6/ Mg_3_(PO4)_2_·22H_2_O/ H_2_O (red), Na-IP6/ Mg_3_(PO4)_2_·22H_2_O/ H_2_O (gray) and their components (purple, green, blue); (**B**): OPLS/ Mg_3_(PO4)_2_·22H_2_O/ H_2_O (red), OPLS/ Mg_3_(PO4)_2_·22H_2_O/ H_2_O (gray) and their components (purple, green, blue) (“pp”: pyrophosphate; “m”: magnesium oxide; *^1^: y-values were multiplied by 0.1 for better illustration).

Figure 4 shows X-ray diffractometry of the cement compositions and their components. In the set cement with Na-IP6, heat-treated TMP hydrate, and H_2_O, only the TMP hydrate peak at 2θ ≈ 30° (magnesium pyrophosphate) remains visible, while magnesium oxide peaks disappear. Similar behavior is observed in the OPLS-containing version of this cement. For the set cement containing Na-IP6, heat-treated cattiite, and H_2_O, some residual peaks, attributed to newberyite, appeared amidst a predominantly amorphous background and are also present in the OPLS-containing counterpart.

**Fig. 5:**
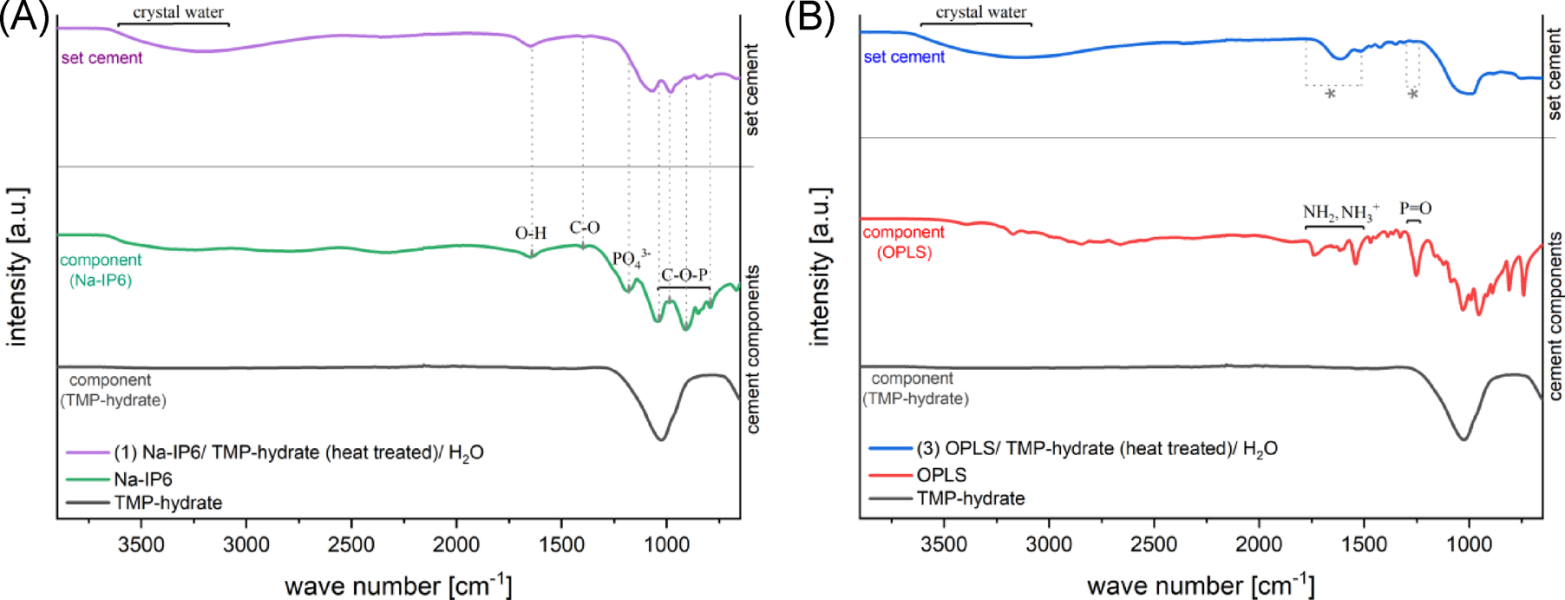
FTIR transmission spectra (incl. FTIR of the reactant powders) in the wave range of 4000 to 650 cm^−1^ of (**A**) the Na-IP6 containing composition (1) (Na-IP6/ TMP-hydrate (heat treated)/ H_2_O) and (**B**) the OPLS containing composition (3) (OPLS/TMP-hydrate (heat treated)/H_2_O).

Figure 5 shows the infrared spectra of cement containing TMP hydrate. FTIR spectra of the congruent cement with cattiite are excluded due to their similarity, with reference data available elsewhere^17^. In OPLS spectra, relevant peaks can be identified, which can be assigned to –NH^3+^ and –NH_2_. –NH^3+^ usually shows absorption just below 1500 cm^−1^ and between 1775 and 1600 cm^−1^, whereas –NH_2_ provokes deformations between 1650 and 1560 cm^−1^^19^. In all OPLS-containing cements, these peaks were levelled out during setting. A P = O peak at 1300–1250 cm^−1^^19^ also vanishes during setting. Sodium phytate shows peaks for C–O stretching (1398 cm^−1^), PO_4_^–3^ (1186 cm^−1^, 496 cm^−1^), and C–O–P vibrations (1038, 986, 850, 793 cm^−1^)^20,21^, most of which disappear in the set cement. An O–H band at 1645 cm^−1^ indicates adsorbed water^22^, and all set cements exhibit a broad band at 3600–3000 cm^−1^ due to crystal water^19^, which can also be explained by the presence of crystal water. The extinction of these peaks suggests that the OPLS and sodium phytate functional groups form coordinative bonds with Mg^2^⁺ in chelate complexes.

**Fig. 6:**
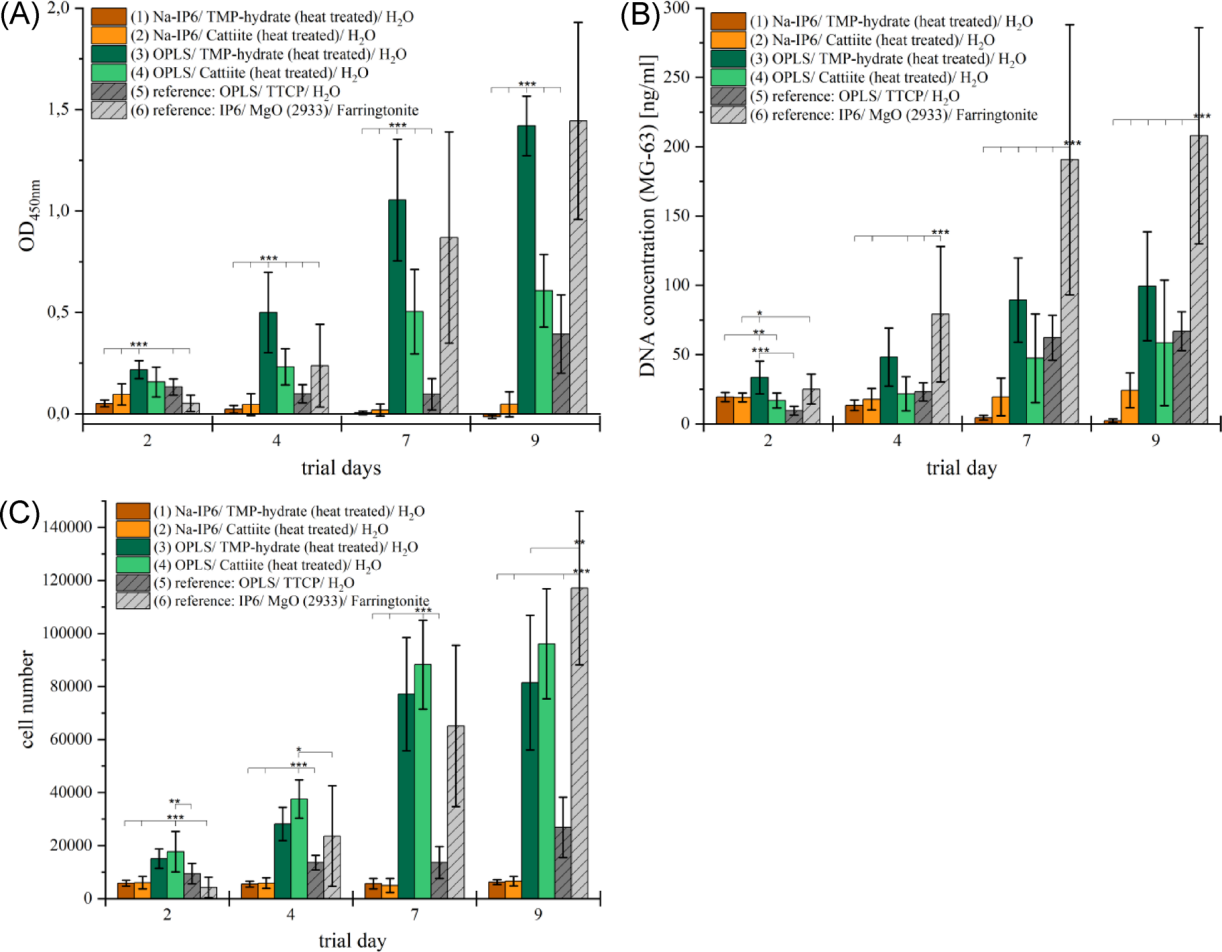
After culturing MG-63 on adhesive cement test specimens (n = 4 each) for 9 days in two independent preparations, measurements were carried out to quantify cell activity and proliferation. (**A**) Photometric absorbance (450 nm) per test specimen in WST-1 assay. (**B**) Determined DNA concentration using a PicoGreen assay. (**C**) Cell number per test specimen. The results of both approaches were included in the calculation of the data presented here. Accordingly, for each assay and cement group, n = 8 (based on independent cement discs; 2 aliquots per disc). *Statistical analysis*: ANOVA was performed separately for each time point, followed by Tukey’s HSD post hoc test. A significance level of α = 0.05 was applied. Only selected significant comparisons are shown for clarity. *p < 0.05; **p < 0.01; ***p < 0.001. OPLS + TMP-hydrate showed significantly higher WST-1 values than all other cements at trial days 2 and 4 (*p *< 0.001). At trial days 7 and 9, both OPLS + TMP-hydrate and reference (6) cement exhibited significantly higher WST-1 values compared to all other groups (*p *< 0.001), with no significant difference between the two. In the PicoGreen assay, OPLS + TMP-hydrate resulted in significantly higher DNA concentrations than all other groups at day 2 (*p *< 0.05 to* p *< 0.001). At days 7 and 9, the reference (6) cement showed significantly higher DNA concentrations than all other groups (*p *< 0.001). At day 4, reference (6) cement also exhibited higher values than all other groups (*p *< 0.001), except for OPLS + TMP-hydrate, which was not significantly different (*p *= 0.079). In the cell count, OPLS + Cattiite consistently showed the highest cell numbers at days 2, 4, and 7. At day 2, all other cements exhibited significantly lower values (*p *< 0.05 to* p *< 0.0001). At days 4 and 7, OPLS + TMP-hydrate, and at day 7, reference (6), showed no significant difference to OPLS + Cattiite. By day 9, reference (6) cement showed the highest values (*p *< 0.0001 vs. all other groups except OPLS + Cattiite). Furthermore, no significant difference was observed between OPLS + Cattiite and OPLS + TMP-hydrate at day 9.

Figure 6 shows results from two independent approaches with MG-63 cells cultivated on n = 4 test specimens of six bone adhesives over nine days. In the WST-1 assay, cements (1) and (2) showed minimal absorption at 450 nm and consistently low cell counts, with the lowest DNA concentrations among all cement. Composition (3) initially had the highest DNA concentration (33.49 ± 11.80 ng/ml) and optical density at 450 nm, which steadily increased to a peak of 1.42 ± 0.15 on day 9, alongside a cell count of 81,455 ± 25,382 (163% of the seeded cells). For composition (4), WST-1 and PicoGreen values were about half those of (3) but showed the highest cell counts until day 7, reaching 96,069 ± 20,743 cells on day 9 (192% of seeded cells). Composition (5) showed stable optical density at 450 nm between days 2 and 7, with a slight increase to 0.39 ± 0.19 on day 9. Cell counts peaked at 26,893 ± 11,358 (54% of seeded cells), the lowest among OPLS-containing cement. The DNA concentration increased sharply between days 4 and 7, reaching 66.88 ± 13.95 ng/ml on day 9. Composition (6) exhibited the highest optical density (1.44 ± 0.49) and cell count (117,098 ± 28,897) on day 9, along with the highest DNA concentration (208.02 ± 78.02 ng/ml).

Confidence intervals (95%) were calculated to assess the statistical reliability of the observed group means across all biological assays (WST-1, PicoGreen, and cell count). All data are based on n = 16 replicates per group. On day 9, group (1) consistently showed the lowest values across all parameters, including absorbance (–0.0138, 95% CI: –0.0182 to –0.0094), DNA content (2.27 ng/well, 95% CI: 1.54 to 3.01), and cell number (6246 cells/well, 95% CI: 5752–6739). In contrast, group (6) exhibited the highest values in both DNA content (208.02 ng/well, 95% CI: 168.27– 247.76) and cell number (117,098 cells/well, 95% CI: 97,150–137,045), while group (3) showed the highest absorbance (1.4196, 95% CI: 1.3415–1.4976). Non-overlapping confidence intervals between several groups suggest statistically distinguishable differences in metabolic activity, DNA content, and cell proliferation.

**Fig. 7:**
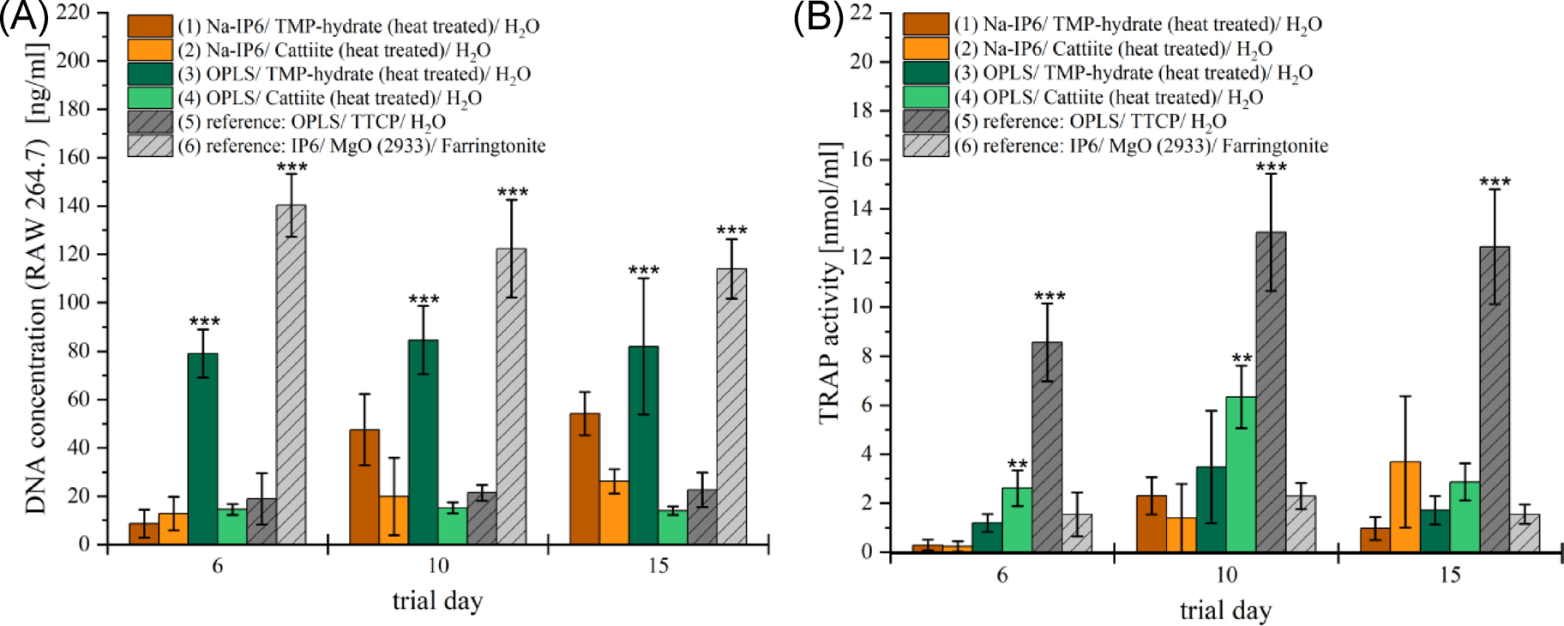
After culturing RAW 264.7 cells on test specimens of six different adhesive cement (n = 4 each) for 15 days in two independent approaches, measurements were performed to quantify cell differentiation and proliferation. (**A**) DNA concentration per milliliter of cell lysate determined using the PicoGreen assay. (**B**) Concentration of pNPs produced per milliliter determined in the TRAP assay cell lysate (TRAP activity). The results of both approaches were included in the calculation of the data presented here. Accordingly, for each assay and cement group, n = 8 (based on independent cement discs; 2 aliquots per disc). *Statistical analysis*: ANOVA was performed separately for each time point, followed by Tukey’s HSD post hoc test. A significance level of α = 0.05 was applied. Only selected significant comparisons are shown for clarity. *p < 0.05; **p < 0.01; ***p < 0.001. Reference (6) cement showed significantly higher DNA content than all other groups at all time points (*p *< 0.001). OPLS + TMP-hydrate showed significantly higher DNA content compared to all other formulations except reference (6) cement (*p *< 0.001). Reference (5) cement exhibited significantly higher TRAP activity than all other groups at all time points (*p *< 0.001). OPLS + Cattiite showed significantly increased values at days 6 and 10 (*p *< 0.01), compared to all other groups except reference (5) cement.

Figure 7 shows the TRAP activity of RAW 264.7 cells cultivated on n = 4 test specimens of six cements over 15 days, with RANKL-containing medium added six times, alongside DNA concentration from a PicoGreen assay. TRAP activity was quantified photometrically by measuring the conversion of p-nitrophenylphosphate (pNPP) to p-nitrophenol (pNP) in the cell lysate. Compositions (1) and (2) had the lowest TRAP activities on day 6, which later increased. Composition (1) peaked at 2.31 ± 0.76 nmol/ml pNP on day 10 before declining, while composition (2) reached 3.68 ± 2.68 nmol/ml on day 15, the second-highest among the formulations. Compositions (3) and (4) also peaked on day 10, with TRAP activities of 3.48 ± 2.30 and 6.34 ± 1.28 nmol/ml, respectively. The DNA concentration remained stable for both: 79.09–84.54 ng/ml for (3), the second-highest overall, and 13.99–15.18 ng/ml for (4). Composition (5) showed the highest TRAP activity, peaking at 13.05 ± 2.39 nmol/ml on day 10, but had low and stable DNA concentrations of 18.97–22.65 ng/ml. Composition (6) had the lowest TRAP activity, peaking at 2.29 ± 0.53 nmol/ml on day 10, but the highest DNA concentrations at all time points, reaching a maximum of 140.28 ± 12.98 ng/ml on day 6.

Confidence intervals (95%) were calculated to assess the statistical reliability of group means across all biological assays performed with RAW 264.7 cells (PicoGreen assay and TRAP activity), based on n = 16 replicates per group. In the PicoGreen assay, group (1) showed a DNA content of 54.23 ng/well on day 15 (95% CI: 49.44–59.02), whereas group (6) reached the highest value with 113.97 ng/well (95% CI: 106.34–121.60). For TRAP activity, group (1) showed 2.31 on day 10 (95% CI: 1.90–2.71), while the highest value was observed in the tetracalcium phosphate (TTCP) reference group (13.05, 95% CI: 11.76–14.34). Non-overlapping confidence intervals across multiple groups and timepoints indicate statistically distinct outcomes in both assays.

**Fig. 8:**
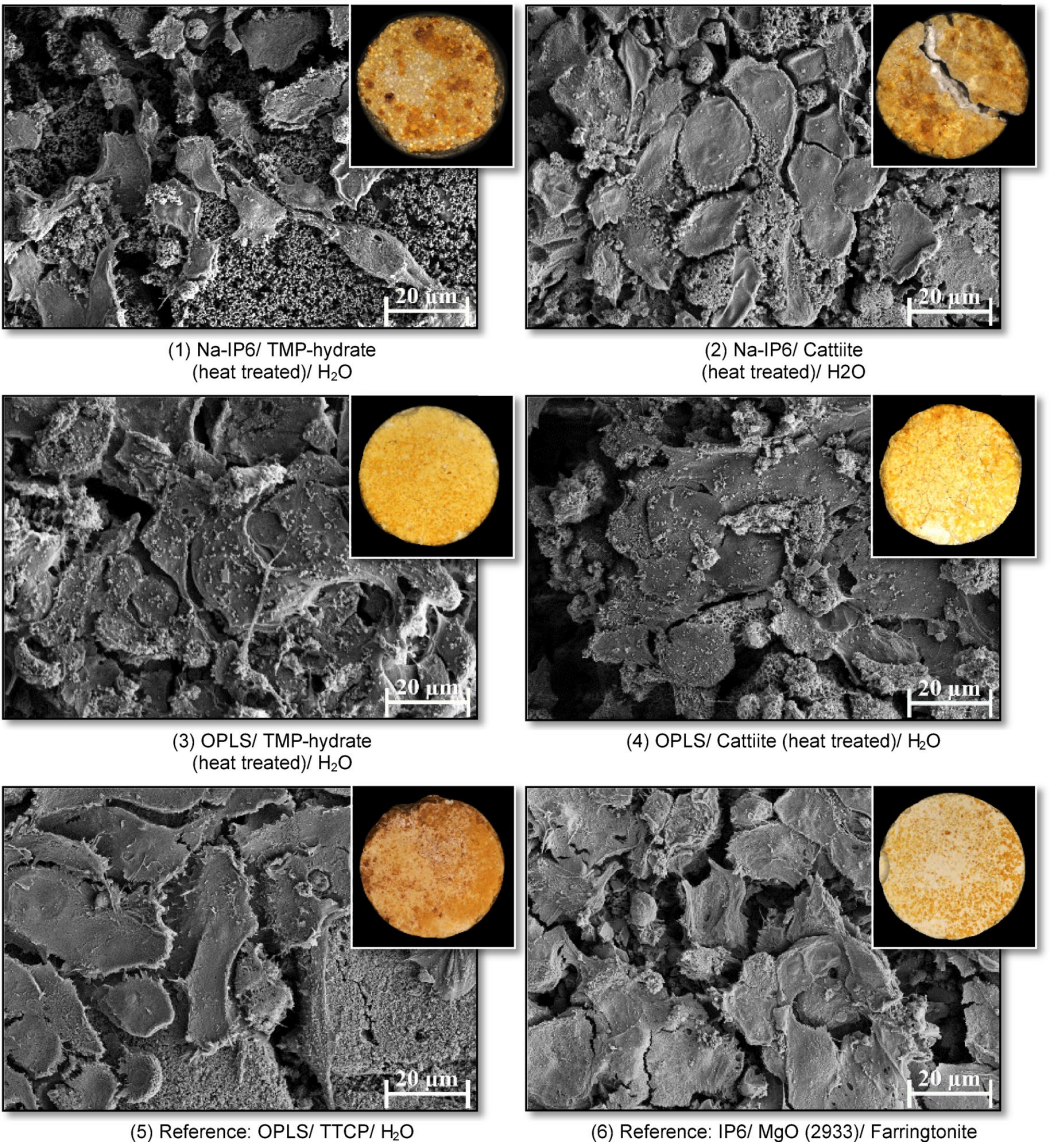
Scanning electron micrographs of cement test specimens of the compositions after 15 days of colonization with RAW 264.7 cells, which were stimulated to differentiate by the regular addition of RANKL. The images were taken at 1000 × magnification. Inlay-figures: Light microscopic images of TRAP-stained cement test specimens of the cement compositions after 15 days of colonization with RAW 264.7 cells, which were stimulated to differentiate by regular additions of RANKL. The test specimens were dried for 7 days after staining and imaged at 20 × magnification. TRAP-positive osteoclasts are shown in bright red to maroon color. The diameter of the viewing aperture is 5 mm in each case.

Figure 8 shows light microscope images of RAW 264.7-colonized adhesives on day 15 of colonization, highlighting TRAP-positive osteoclasts in bright red to maroon. Different compositions exhibited distinct cell growth patterns and color changes.

In composition (1), small cell clusters were larger and more numerous, with maroon areas dominating by day 15. Composition (2) showed strongly colored cell clusters from day 6, developing into dense, dark clusters and maroon areas by day 15. Composition (3) had a yellow base with brownish clusters from day 6, which expanded into larger colored areas by day 10, fading somewhat by day 15. Composition (4), though highly reflective, followed a similar pattern to (3), with brownish clusters reducing to spot-like colorations by day 15. Composition (5) developed chestnut-brown areas at the edges by day 6, which expanded and covered the specimen by day 15, showing strongly colored edges. Composition (6) primarily exhibited light yellowish areas with small dark cell accumulations visible only on days 10 and 15.

Figure 8 shows electron micrographs of RAW 264.7 cell-colonized adhesives after 15 days of RANKL-stimulated differentiation. On composition (1), round, undifferentiated cells (~ 10 µm) were present on day 6, forming 20–30 µm fusing cell islands by day 10, which increased in size and number by day 15. Composition (2) exhibited similar growth, with cells reaching up to 40 µm by day 15.

Compositions (3–6) showed cracked surfaces. Composition (3) was nearly fully colonized with extracellular matrix and globular particles, whereas composition (4) displayed larger, more spread cells, up to 50 µm by day 10. Composition (5) had sparse colonization but showed relatively large, spread cells (up to 60 µm) already on day 6, indicating changes in morphology typically associated with differentiation. Composition (6) was densely colonized with cells (10–30 µm, occasionally > 50 µm), with no significant changes over time.

**Fig. 9:**
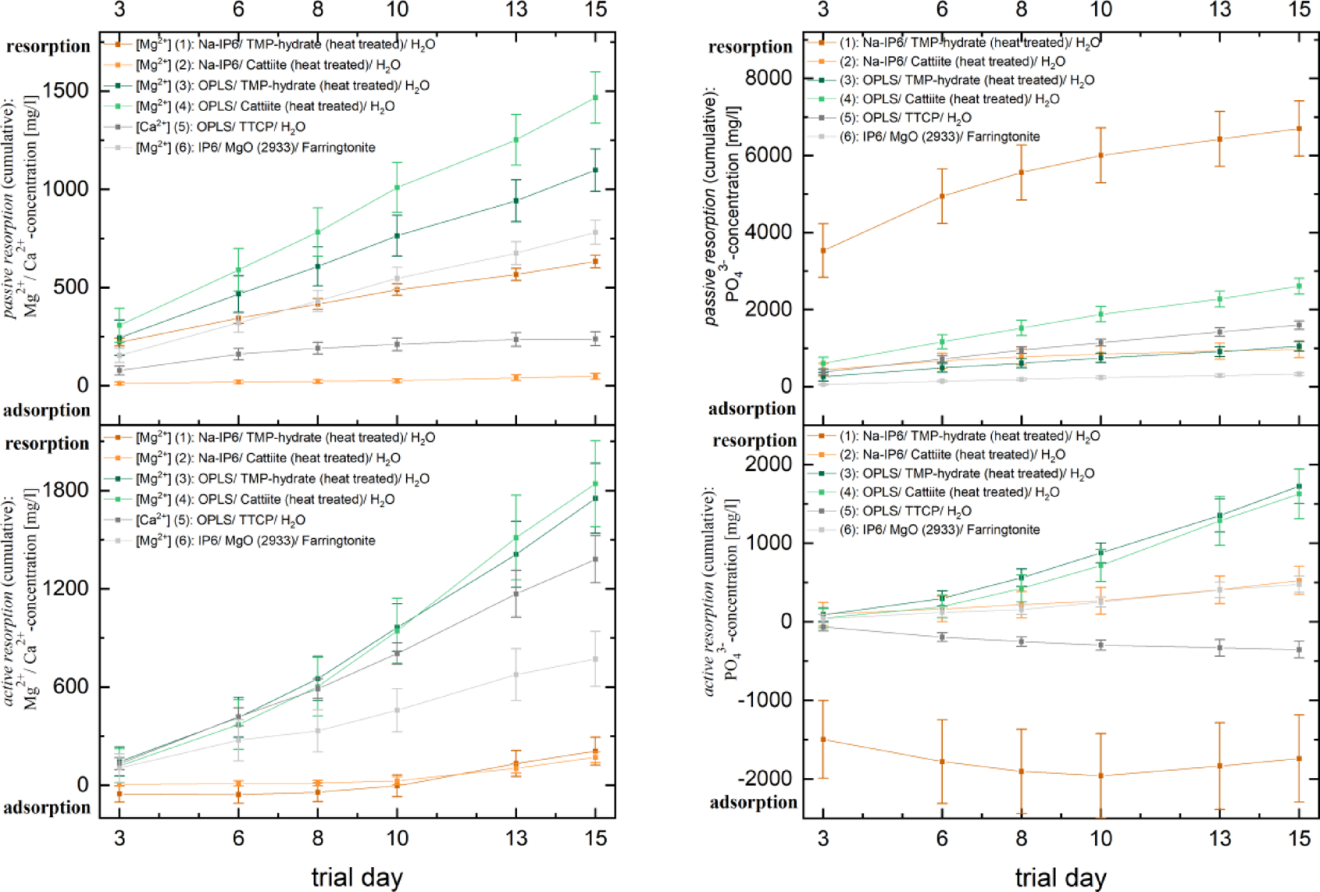
During the cultivation of RAW 264.7 on adhesive cement test specimens (n = 4 each) for 15 days in two independent batches, supernatants of the culture medium were preserved on test days 3, 6, 8, 10, 13 and 15 and the Mg^2+^/ Ca^2+^/ PO_4_^3-^ concentration was determined using ICP-MS (total resorption). The same procedure was carried out on uncolonized test specimens (n = 4 each) (passive resorption). The resorption by RAW 264.7 (active resorption) resulted from the difference between total and passive resorption. Left: Cumulative passive (top) and active (bottom) resorption in terms of Mg^2+^/ Ca^2+^: Cumulation and mean values on the respective test day. Right: Cumulative passive (top) and active (bottom) resorption in terms of PO_4_^3-^: Cumulation and mean values on the respective test day. The results of both approaches were included in the calculation of the data presented here.

Figure 9 shows cumulative mean concentrations of Mg^2^⁺ and PO₄^3−^ in the supernatant of culture media for RAW 264.7 colonized and uncolonized test specimens, highlighting total, active, and passive resorption as well as adsorption processes. For simplicity, Ca^2^⁺ values are shown only for composition (5), and individual non-added values are omitted. The cumulative concentration (*c*_*cum*_) on day n, representing the sum of concentrations from previous days, was used to calculate the cumulative standard deviation (*σ*_*cum,n*_): σ_cum, n_ $=\sqrt{{\upsigma }_{1}^{2}+{\upsigma }_{2}^{2}+\dots +{\upsigma }_{n}^{2}}$.

Compositions (2) and (5) showed minimal passive resorption. Mg^2^⁺ concentration in composition (5) was below the blank medium, indicating slight adsorption without cell activity. From day 10, composition (2) exhibited increasing cell activity, reaching ~ 171.42 mg/l active resorption by day 15. Compositions (1), (3), (4), and (6) released significantly more Mg^2^⁺ passively, with composition (4) reaching the highest cumulative concentration (1466.18 mg/l). Composition (1) initially adsorbed Mg^2^⁺ but transitioned to active resorption from day 8. Compositions (3), (4), and (6) consistently resorbed Mg^2^⁺, with composition (4) achieving the highest cumulative resorption (1841.42 mg/l). Composition (1) showed high passive PO₄^3−^ resorption initially (~ 7000 mg/l), with cells adsorbing 1496.93 mg/l by day 3. Although 1740.35 mg/l PO₄^3^⁻ was actively adsorbed, ~ 5000 mg/L still entered the medium due to passive resorption. In composition (5), cells adsorbed 353.54 mg/l PO₄^3−^, while passive resorption released 1597.64 mg/l, resulting in ~ 1000 mg/l ions entering the solution. Other cements showed combined passive and active PO₄^3−^ resorption. Composition (4) had the highest passive release (~ 2610.27 mg/l), while compositions (2) and (3) passively released ~ 1000 mg/l. Active resorption was highest in composition (3) (1723.70 mg/l), with compositions (2) and (6) each resorbing ~ 500 mg/l.

## Discussion

The aim of this study was to develop bone adhesives based on magnesium phosphates and organophosphates for clinical use. Phosphoserine was used as a bioadhesive component, while sodium phytate was investigated as a novel organophosphate to enhance adhesion through its high density of functional groups. Magnesium phosphates replaced conventional calcium phosphates to improve both adhesive and biological properties. Thermally treated, partially amorphous magnesium hydrates were employed to increase reactivity and promote a homogeneous, hydrostabile matrix. In addition to setting behavior and strength, the focus was on biological performance—especially since such material combinations have not yet been studied in cell-based models within the context of bone adhesives.

The classic reaction mechanism of magnesium phosphate cements is based on a continuous dissolution/precipitation reaction in which dissolved phosphate anions and magnesium-containing cations form an amorphous gel that leads to crystallisation through thickening^16,23^. Christel et al. (2015) proposed an additional chelate complex formation between phosphate groups of phytic acid and Mg^2^⁺ ions for a cement of farringtonite, MgO and IP6^24^, based on similar mechanisms in calcium phosphate cements^25^. Functional groups of other organophosphates such as OPLS could also form chelate complexes with Mg^2^⁺-^17^ or Ca^2^⁺ ions^26,27^. For the novel compositions (1) and (2) in this work, chelate complex formation would also be conceivable. The water of crystallisation detected by FTIR shows hydration, while the disappearance of certain P-OH vibrations and phosphoric acid ester bands indicates reactions of these groups, possibly through coordinative bonds to Mg^2^⁺ ions in solution (see Fig. 5).

On the heat-treated TMP hydrate, residual MgO peaks can still be recognised by X-ray diffraction, which disappear during the reaction (see Fig. 4). MgO could quickly go into solution and react to form Mg(OH)_2_, which dissociates to form Mg^2^⁺- and two OH- ions, raises the pH and provides Mg^2^⁺ for complex formation^23^. After the reaction, a peak for magnesium pyrophosphate remains, and the chelation of metal ions by pyrophosphates is also conceivable. In the diffractogram of originally cattiite, residual peaks for newberyite are visible after the reaction, which suggests a continuous dissolution/precipitation reaction in addition to the chelate complex formation. The formation of newberyite only in composition (2) could be due to the higher Na-IP6 content, analogous to the results of Christel et al. (2015), who also found more newberyite in the cement with increasing phytic acid concentration^24^.

In addition to the chelate complex formation with Mg^2^⁺ during the setting reaction, coordinative bonds to Ca^2^⁺ of the inorganic bone are also conceivable, which could explain the adhesive properties of the cements. The –NH₃⁺ residues of the amino acids (hydroxy)proline and (hydroxy)lysine contained in type I collagen^28^ could also facilitate such bonds.

Farrar (2012) points out that heat development can be problematic when curing bone cement^29^. However, the adhesives presented reach a maximum temperature well below body temperature, so that neither tissue damage nor restrictions with temperature-sensitive medications^30^ are expectable. The pH value is also decisive: an acidic environment can drive osteoblasts to apoptosis^31^, release proinflammatory cytokines^32^ and inhibit mineralization^33^. Brandao-Burch et al. (2005) found optimal mineralisation at pH 7.4 and complete inhibition at pH 6.9^33^. Although the pH values of most compositions remained below 6 (see Fig. 3), this may be buffered in vivo^34,35^. Still, near-neutral pH is desirable to support osteogenesis.

The results on mechanical parameters show that the decisive factor of adhesive strength can be very high regardless of the compressive strength (see Fig. 2). All tested compositions exceeded the frequently cited minimum bond strength of 0.2 MPa required by Weber and Chapman (1984)^18^. Composition (5) is considered by the authors to be a derivative of the bone adhesive Tetranite®, which appears to be close to approval. The formulation is taken from US-patent 8,273,803 (“Table 1, COMPOSITION 1B”)^36^. However, this and the second reference composition (6) show a significantly lower adhesive strength than the new formulations presented here (see Fig. 2). Composition (1) initially exhibited approximately 3.9 times and composition (2) 2.6 times the adhesive strength of the reference composition (5) on the bone. Composition (3) initially achieved approximately 3.5 times the adhesive strength of composition (6) on the bone and composition (4) 3.1 times the adhesive strength. Even with ageing of the bond, the difference in bond strengths remains clearly visible. The observed interplay between adhesive strength and compressive strength reflects a classic materials design trade-off. While high compressive strength is generally associated with increased internal cohesion, it may reduce the material’s ability to achieve strong interfacial adhesion due to diminished wettability or tack. A similar inverse tendency between cohesion and tack is noted in a standard textbook on adhesive technology by Habenicht et al.^37^, although not in the specific context of cementitious systems. In our materials, a slight excess of organophosphates appears to enhance adhesion but reduces compressive strength marginally. Clinically, this trade-off is acceptable, as even small changes within the 0.5–4.5 MPa adhesion range can greatly affect performance, while similar shifts in compressive strength are less critical. Still, sufficient cohesion is essential to avoid cohesive failure, which would compromise the adhesive regardless of its interfacial performance^38^.

Furthermore, biological analyses were performed to assess the cellular compatibility of the different compositions.The cell biological results will be discussed in the following sections:

A general observation was, that mineral-organic cements based on Na-IP6 (compositions (1) and (2)) were found to be insufficiently biocompatible with osteoblast-like MG-63 cells. This could be due to the high phytic acid content and high phosphate release, which leads to cytotoxic effects^39^. The pH of these compositions was more acidic during curing and washing, which could negatively affect cytocompatibility^40^. In contrast, MG-63 cells proliferated better on the OPLS-containing compositions (3) and (4) and achieved significantly higher cell activity and numbers compared to the reference composition (5). According to the literature, the addition of OPLS promotes proliferation and differentiation of bone cells and improves osteoconductivity^41–44^. All in all, compositions (3) and (4) showed promising biocompatible properties that are in line with previous studies and also compare favourably with established products in terms of their adhesive properties.

Moreover, our results could indicate a higher biocompatibility compared to calcium phosphate and OPLS-based compositions (5). Ewald et al. (2011) reported comparable or better cytocompatibility of magnesium phosphate cement compared to calcium phosphate cement with higher cell proliferation, which could be explained by the release of Mg^2^⁺ ions^45^. High Mg^2^⁺ concentrations promote osteoblast proliferation, differentiation and activity^46–48^, while high Ca^2^⁺ concentrations are cytotoxic^49^.

In the WST-1 assay, composition (3) showed twice as high values as composition (4), although similar numbers of cells were counted on (4), indicating a stronger stimulation of metabolic activity on composition (3). At day 7, composition (3) showed higher WST-1 and DNA values despite a lower number of adherent cells compared to composition (4). This apparent contradiction may be attributed to material–cell interactions affecting metabolism and attachment independently. Elevated mitochondrial activity per cell could explain the stronger WST-1 signal, while total DNA measurements also include contributions from non-adherent or lysed cells. A similar discrepancy between cell number and DNA concentration was observed in composition (5), potentially due to cell death during proliferation, with DNA still detected by the PicoGreen assay^50,51^. Overall, composition (3) as well as reference composition (6) showed excellent cytocompatibility towards MG63 cells and even outperformed reference composition (5)^3^. The relatively high standard deviations observed in the cell viability assays (see Fig. 7) may be attributed to variations in the cement surface topographies and possible batch-to-batch differences during specimen preparation. The cements were mixed manually with a spatula on a glass plate and have a short working time, which—while clinically beneficial—can limit processing precision and lead to slight inconsistencies in surface structure and elution behavior.

In addition to MG-63 cell experiments, osteoclasts were examined by direct colonization to assess their interaction with the cements. RAW 264.7 cells differentiated with RANKL show strong differentiation^52,53^, especially with reference composition (5), the only calcium phosphate cement that released calcium ions. TRAP activity was highest in reference composition (5), while DNA concentration remained low. Electron microscopy revealed large, spread osteoclasts, indicative of advanced cell differentiation. Calcium ions play a key role in the differentiation of osteoclasts, and their high concentration could lead to apoptosis^54–56^, which could explain the decrease in measured values after day 10. Magnesium ions, on the other hand, promote proliferation but inhibit differentiation of osteoclasts, which is consistent with the observations with magnesium phosphate cements^48,57–59^. For samples of compositions (1) and (2), neither TRAP activity nor DNA concentration could be measured at the beginning. However, on test day 15, composition (1) showed an approximately 2.4-fold and composition (2) a 1.2-fold DNA concentration compared to reference composition (5). TRAP activity also increased and reached its maximum for composition (1) on test day ten, while for composition (2) it increased over the entire course of the test and reached the highest value among the magnesium phosphate-containing cements analyzed on test day 15, which corresponded to about 30% of the value calculated for composition (5).

Qualitative tests largely confirmed these results: On no magnesium phosphate-containing cement test specimen were similarly large and intensely colored areas visible in the TRAP staining as in composition (6). Test specimens of composition (2) showed weaker but still intensely colored clusters on test days 10 and 15. SEM images of test specimens from composition (1) showed only a few cells with undifferentiated morphology at the beginning, which proliferated over time and gradually developed features suggestive of differentiation. Test specimens of composition (2) were colonized by large, spread cells with diameters of up to 40 µm, indicating a morphology consistent with differentiated osteoclasts. The results indicate that RAW 264.7 cells on test specimens of the NaIP6-containing compositions (1) and (2) proliferate slowly but to a considerable extent and differentiate into osteoclasts under the influence of RANKL. Compared to RAW 264.7 and osteoclasts differentiated from it, they demonstrate a higher cytobiocompatibility in cell experiments than compared to MG-63 cells. Antineoplastic properties of phytic acid^60–62^ could possibly have an influence on the vegetation of the osteosarcoma cell line MG-63. A higher resistance of RAW 264.7 cells to potentially cyototoxic high phosphate concentrations^39^ is also conceivable. When comparing the Na-IP6-containing formulations with each other, it is noticeable that the cells in composition (1) proliferated more clearly to the end, while they differentiated to a higher degree in composition (2) (composition (1) to (2) on test day 15: 2.1 times the amount of DNA, but only 26% of TRAP activity). SEM images and TRAP staining support this finding.

Composition (1) stands out due to an excessive passive release of phosphate ions into the medium, which cannot be equalised despite active adsorption by the cells (see Fig. 9). The high phosphate concentration could inhibit osteoclast differentiation^59^. Magnesium ions are also passively released into the medium to a significantly higher extent by composition (1) (see Fig. 9), which could promote cell proliferation and in turn inhibit their differentiation^48,58^. Iron-dependent mitochondrial biogenesis is central to osteoclast differentiation and activity^63^. Phytic acid can form strong chelate complexes with iron^64^, which could suggest an inhibition of osteoclast differentiation^65^. The results for composition (1) and (2) partially contradict this, as considerable differentiation was demonstrated here. Since Na^+^ or Mg^2+^ bonds are coordinately bound by phytic acid, iron complexation does not appear to play a decisive role here. However, in the case of cements containing IP6, such as composition (6), iron complexation could be relevant.

Composition (6) showed its maximum TRAP activity on test day 10, comparable in level to composition (1), and generally the highest DNA concentration at all measurement times among all cements. TRAP-stained test specimens were slightly yellow in color and the SEM showed colonization with rather small cells (10–30 µm, occasionally > 50 µm). Taken together, the results indicate a rapid proliferation of RAW 264.7 cells with moderate differentiation. In this context, Arriero et al. (2012) postulated that IP6 inhibits osteoclastogenesis from both RAW 264.7 and human cells^66^. However, the assumption that phytic acid alone is correlated with reduced differentiation is contradicted by the observations of Meininger et al. (2017), who observed a clear differentiation of the cells in calcium phosphate cements containing phytic acid compared to RAW 264.7, albeit only via TRAP staining^8^. In addition to phytic acid, the predominant farringtonite phase of the cured composition (6)^10^ could have an influence on cell development. Schröter et al. (2024) found a strong colonization of farringtonite granules with osteoblasts and osteoclasts in in vivo experiments in sheep^67^. However, Ostrowski et al. (2015) found a strong proliferation of RAW 264.7 cells for trimagnesium phosphates regardless of their crystal structure, but no evidence of differentiation into osteoclasts^47^. The lack of differentiation into osteoclast-like cells is only associated with the high magnesium content^47^. The strong Mg^2+^ degradation in composition (6), which promotes osteoclast proliferation while inhibiting differentiation^48,58^, could also be the reason for the discussed relationship between proliferation and differentiation. Despite the discussion, it should be mentioned at this point that the differentiation of RAW 264.7 into multinucleated osteoclast-like cells was observed in all cements analyzed, regardless of the nature of the magnesium phosphate or the content of phytic acid or phytate.

With regard to Mg^2+^ degradation and the influence on cell development described in the literature, compositions (3) and (4) should be emphasised in this context. Their recorded Mg^2+^ degradation is clearly evident, although here and at the same time the results paradoxically indicate greater differentiation with relatively lower proliferation. Thus, considerable TRAP activities are determined, which in their maximum on test day 10 nevertheless corresponded on average to only approx. 27% or approx. 49% of the value of the reference composition (5), which is also OPLS-based. These findings should be considered in light of previous reports on the concentration-dependent effects of extracellular Mg^2+^ on osteoblasts. Lu et al.^68^ observed increased ALP activity, osteocalcin expression, and mineralization in primary human osteoblasts at Mg^2^⁺ concentrations between 0.5 and 2 mM, while higher levels (≥ 4 mM) reduced these markers. He et al.^69^ similarly reported rising ALP and osteocalcin levels between 1 and 3 mM, along with enhanced gap junction communication. However, both studies used soluble magnesium salts in standard culture systems and are not directly transferable to solid materials. In the present adhesives, magnesium is part of a crystalline phase and released gradually, depending on solubility and environmental factors. Although higher Mg^2+^ content may contribute to the observed cellular effects, the actual ion concentrations in the microenvironment remain unknown.

While a multiple of the DNA concentration was recorded in the samples from composition (3) compared to composition (5), samples from composition (4) showed slightly less DNA content. This is also reflected in the qualitative evidence, but especially in the SEM images. These observations indicate a considerable differentiation of RAW 264.7 cells when colonizing OPLS-containing magnesium phsophate cements, especially in composition (4). At the same time, a strong proliferation of cells can be assumed on composition (3). Both cements are more actively resorbed by cells with regard to both magnesium and phosphate ions than all other cement formulations investigated, which may also indicate the advanced differentiation of the cells. The simultaneous passive release of comparatively high amounts of magnesium ions into the medium contradicts the assumption that high extracellular Mg^2+^ concentrations inhibit differentiation^48,58^. This cannot be confirmed with regard to OPLS-containing adhesive cements.

It is conceivable that OPLS mediates the adhesion of RAW 264.7 cells to the cement test specimen and subsequently increases their differentiation into osteoclasts in the sense of an imitation of the bone matrix protein osteopontin, which has been described previously^70^. Most bone cells, such as osteoclasts and their precursors, bind to osteopontin, which not only mediates adhesion to the bone matrix, but also stimulates differentiation and activity^71^. This could also explain the promotion of adhesion and early proliferation and activity of osteoblast-like MG-63 cells described above.

In a rat study, Schneiders et al. (2007) reported that the addition of phosphoserine to calcium phosphate cements accelerates bone remodelling, particularly in the early stages of healing^70^. TRAP-positive cells with phosphoserine appeared earlier and in greater numbers in the vicinity of the cements^70^. Reinstorf et al. (2006) also found that OPLS supplementation strongly promotes monocyte activation and accelerates bone remodelling in vivo without impairing osteoclastogenesis^44^. Ogawa et al. (2006) identified L-serine as essential for RANKL-induced osteoclastogenesis of RAW 264.7^72^. Verma et al. (2018) also reported that phosphoserine is closely associated with the fusion of osteoclast precursors into multinucleated osteoclasts^73^. Specifically, the higher proportion of OPLS in composition (4) compared to composition (3) may have stimulated the cells in favour of differentiation and against proliferation. Undifferentiated cells are generally more prone to proliferation than differentiated cells, whereby proliferation even stops with final differentiation^74^.

This study has several limitations that should be acknowledged. First, MG-63 cells were used as an osteoblast-like model for biocompatibility screening. While these cells are widely accepted in material testing due to their reproducibility and robust growth, they are derived from an osteosarcoma and do not fully replicate the behavior of primary human osteoblasts. Future studies should therefore include primary osteoblasts or mesenchymal stem cells to validate these results under more physiologically relevant conditions.

Second, osteoclast differentiation was primarily assessed by TRAP activity, which, although widely used, reflects only one aspect of the differentiation process. Other methods such as RT-PCR or mineralization staining could provide further insights. However, when working with porous cementitious materials, interactions between the material and staining or lysis reagents—as well as background absorption and dye uptake—may interfere with reliable quantification, limiting the applicability of certain assays. Future studies might address these challenges by using adapted protocols or indirect model systems.

Third, although all cement samples were pre-incubated in culture medium for 24 h to minimize acute cytotoxic effects, the eluates from this pre-incubation step were not separately tested. Testing such eluates could help distinguish between transient chemical effects (e.g., pH shifts or early ion release) and material-induced cytotoxicity, particularly relevant for materials applied in unset or pasty form. While Klimek et al. (2016)^75^ focused on correcting ion-depleted extracts, their work underscores the importance of accounting for ion adsorption and medium composition when assessing cytotoxicity of high-surface-area ceramics.

Fourth, the study was limited to in vitro experiments. While the results provide important insights into chemical behavior, adhesive strength, and cellular responses, in vivo studies are essential to evaluate the performance of the materials under physiological conditions, including tissue integration, biodegradation, and systemic tolerance. Future work should therefore include animal models to further assess the clinical potential of the developed bone adhesives.

## Conclusion

In this study, we developed hydrolytically stable mineral-organic bone adhesives based on organophosphates (sodium phytate and O-phospho-L-serine) and amorphous magnesium phosphates. This enabled, for the first time, comprehensive biological evaluation of these cements. Among all tested formulations, the combination of O-phospho-L-serine (OPLS) with heat-treated magnesium phosphate hydrate (composition (3)) showed the most favorable balance of adhesive performance, mechanical stability, and cytocompatibility. In contrast, sodium phytate-based formulations, while initially strong in adhesion, exhibited significantly reduced compatibility with osteoblastic cells and are therefore less suitable for further development. Overall, our results identify the OPLS/MgP-based formulation as the most promising candidate for future in vivo validation as a resorbable bone adhesive with cell-interactive potential.

## Materials and methods

### Powder and sample preparation

To prepare the powder synthesis of raw cattiite powder, a Büchner funnel covered with filter paper was placed in an Erlenmeyer flask and connected to the Labport® N 840.3 FT.18 vacuum pump (KNF Neuberger, Freiburg). 50 ml Na_3_PO_4_·12H_2_O solution (0.4 M) was mixed with 50 ml MgCl_2_·6H_2_O solution (0.6 M) in a beaker, transferred to the funnel and vacuum drawn. The precipitate was rinsed with 500 ml ultrapure water and 200 ml acetone. The process was repeated several times and the powder obtained was stored for 24 h at 37 °C in the UN30 drying oven (Memmert GmbH + Co. KG, Schwabach, Germany). Finally, the powder was heated to 100 °C for 200 min in the high-temperature oven C 19 (Nabertherm GmbH, Lilienthal, Germany). After a holding process of 30 min, the temperature was increased to 400 °C in 200 min, which was maintained for 6 h. The oven cooled down to room temperature. The commercially purchased Mg_3_(PO_4_)_2_·x H_2_O raw powder was heat-treated according to the same protocol.

As part of the raw farringtonite powder synthesis, 0.6 mol MgHPO_4_·3H_2_O and 0.3 mol Mg(OH)_2_ were sintered for 5 h at 1100 °C in the Vecstar sintering furnace system (Oyten Thermotechnik GmbH, Oyten, Germany). After manual mortaring, the powder was sieved to ≤ 355 µm and ground in a planetary mill PM400 (RETSCH GmbH, Haan, Germany) for 1 h at 200 rpm.

To prepare the tetracalcium phosphate (TTCP) raw powder, 12.1 mol CaHPO_4_ and 11.5 mol CaCO_3_ were mixed in a plow mixer for 60 min and sintered in Alsint crucibles for 5 h at 1500 °C. Finally, the powder was sieved to ≤ 125 µm.

To produce all cement, the required quantities of powder were weighed and homogeneously mixed and the dry mass was placed on a glass plate. After adding ultrapure water, all components were quickly mixed using a spatula while constantly squeezing against the glass plate. Depending on the application, the cements were either bonded directly or transferred to silicone moulages. To develop the new cement presented here, defined quantities of heat-treated TMP hydrate or cattiite were combined with variable quantities of phytic acid as part of numerous systematic test series. Cements with phytic acid were feasible and had adhesive properties, but were not pursued further due to their low water resistance. Later, cements were produced from TMP hydrate or cattiite with sodium phytate or OPLS powder and ultrapure water. The target parameters with regard to the cement properties were an application time of 2.5 min via a syringe, dimensional stability after 2.5 min at the latest and a final setting time of no more than 30 min.

### Cement composition and mixing protocol

The following cement formulations were developed and analyzed as part of the research efforts presented here (see Table 1). The table also lists the references used. Reference (6) was selected as it represents a widely recognized and promising mineral-organic bone adhesive formulation in the literature, based on phosphoserine and calcium phosphate (TTCP/α-TCP), as outlined in US Patent 8,273,803^36^. It serves as a benchmark in the field. Reference (5) is from Brückner et al. (2019)^10^ and is interesting for two reasons: it uses phytic acid as the organic component (rather than phosphoserine), providing a comparison to our sodium phytate-based formulations. Additionally, it is based on magnesium phosphate, which is less common in bone adhesives, and since our formulations also use magnesium phosphate, this reference offers a valuable comparison.

**Fig. 10:**
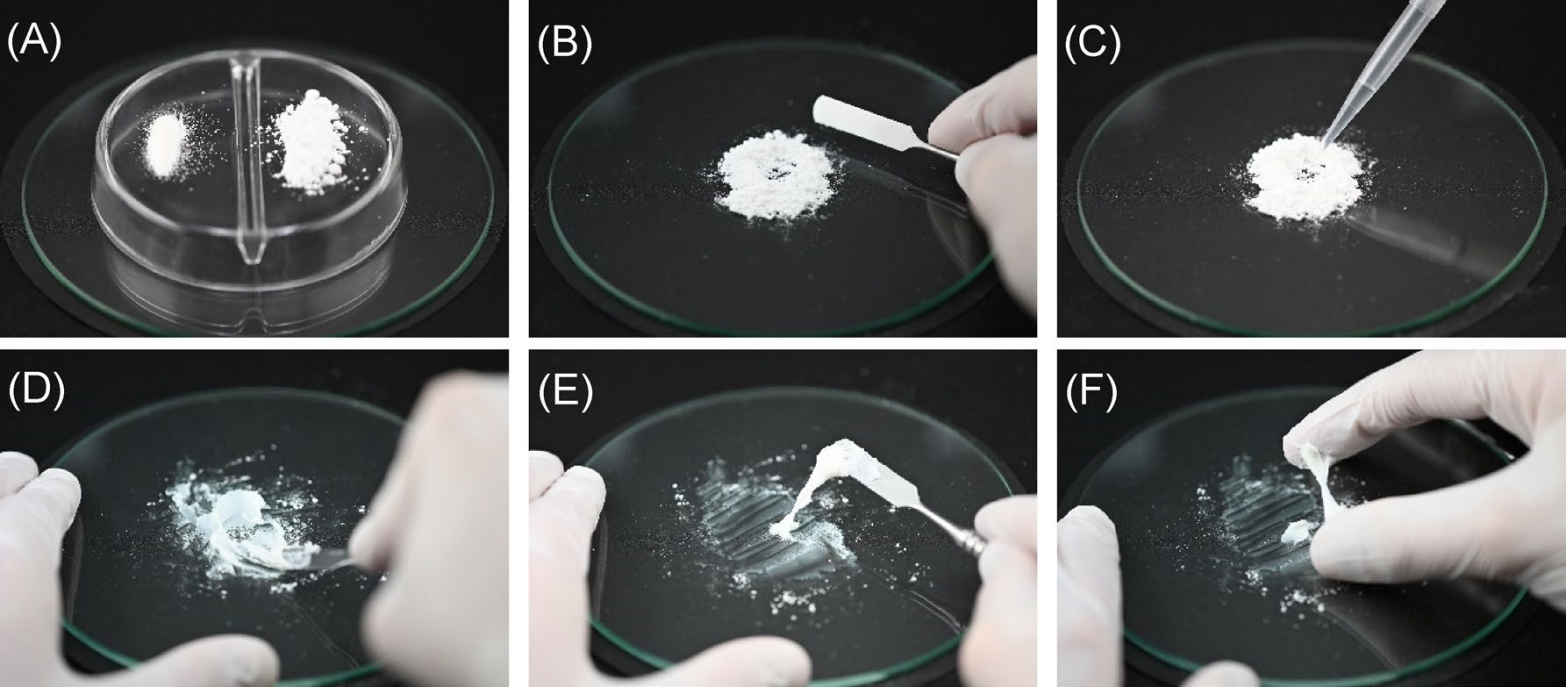
Exemplary procedure of a cement mixing: (**A**) raw powder (here OPLS, heat-treated TMP hydrate); (**B**) mixing of the dry mass; (**C**) addition of water; (**D**) mixing by continuous squeezing against a glass plate; (**E**, **F**) demonstration of cohesion, adhesion, and viscosity. The cement can be transferred to a syringe and applied at this stage.

The cement mixing process is shown as an exemplary illustration in Fig. 10.

### Physicochemical characterization

The cements and cement constituents were analyzed using the D8 Advance X-ray diffractometer (Bruker, Billerica, United States). Monochromatic CuK α radiation was used at a diffraction angle of 2 θ, an accelerating voltage of 40 kV and an emission current of 40 mA within an angular range of 10–70° with a step rate of 1.2 s/step and a step size of 0.02°. The software products DIFFRAC.SUITE, DIFFRAC.COMMANDER, DIFFRAC.EVA V5.1 and DIFFRAC.TOPAS (Bruker Corporation, Billerica, United States) were used to display, evaluate, analyze and quantify the diffraction patterns of the X-ray diffractograms and the reference patterns were taken from the database of the International Centre for Diffraction Data (PDF-2, 1996)^76^. Representative data from one specimen per composition are shown in the result section. The Nicolet™ 10 FT infrared spectrometer (Thermo Fisher Scientific GmbH, Dreieich, Germany) was used to perform the FTIR in a spectrum from 3900 to 640 cm^-1^. The presented spectra in the result section are representative of one measurement per composition or powder. The temperature during the setting process was recorded using the Voltcraft K202 data logger thermometer (Conrad Electronic SE, Hirschau, Germany). The pH evolution was monitored using the pH/Ion Meter SevenDirect SD50 (Mettler-Toledo GmbH, Greifensee, Switzerland), based on three independent runs per composition. The cement samples were quickly transferred into a silicone mold after mixing, and a conforming pH electrode was inserted.

### Mechanical testing

Bovine femora were sourced from a local butcher (Deppisch, Greußenheim, Germany) the day after slaughter. The bones were by-products of routine meat production, and no ethical approval was required for their use in this study. Epiphyses were separated from the diaphysis using a handsaw, and the diaphysis was divided into segments. Muscle, tendons, periosteum, blood vessels, and marrow were removed. Fragments were shaped into rectangular prisms (20 mm × 10 mm × 5 mm) using a Metaserv® 3000 disc grinder (500 rpm, water-cooled, P80 SiC sandpaper). Cylindrical specimens (2.5 mm^2^ x π × 5 mm) were machined on a Robling 800 Stfl precision lathe (2000 rpm). The process was completed within 24 h, with intermediate and final products stored at 5 °C in 1 × PBS solution (pH ~ 7.4).

To determine the bonding strength, the mixed cements were applied to the cylindrical and cuboid parts to be joined using a syringe and these were bonded together in the centre with the aim of achieving the narrowest possible cement joint. Excess cement was removed. The samples were then stored in a water bath at 37 °C and 100% humidity and tested 20 min after the start of the mixing process. Bone test specimens were stored in PBS solution and removed before bonding. The surface was dried with compressed air for 30 s. For each combination of bonded material and each measurement time point, a sample body of n = 10 was selected for each cement. The bond strength was finally determined with the Z010 universal testing machine using the testXpert® II testing software (both from ZwickRoell GmbH & Co. KG, Ulm, Germany). A 10 kN load cell was used to measure from a preload of 1 N at a test speed of 1 mm/s. The sample was fixed against the wall of the test fixture using a screw with the cuboid test specimen so that only the cylindrical test specimen protruded through a metal recess. The test device is established at the institute and has been described elsewhere^17,77^. To determine the compressive strength of the adhesive cements, they were stored in silicone moulds after mixing as described above. This resulted in cement cuboids measuring 6 mm × 6 mm × 12 mm. For each cement and time point, *n* = 10 specimens were tested. The compressive strength was tested on the testing machine with the settings described above by compressing them with a punch until a significant drop in force or a test specimen deformation of 20% occurred.

The fracture behaviour was analyzed using scanning electron microscopy. Bone test specimens are not permitted at the institute due to the residual moisture. In order to avoid damage to the device, adhesive joints were analyzed in the bonding test of calcium-deficient hydroxyapatite (CDHA) test specimens used. For the production of CDHA test specimens, Teflon-milled molds were used, containing rectangular prisms measuring 20 mm × 10 mm × 5 mm and cylinders with dimensions of (2.5 mm)^2^ × π × 5 mm. A negative mold was cast using addition-curing vinyl polysiloxane. These molds were subsequently used as templates for the CDHA test specimens. The CDHA was synthesized from α-tricalcium phosphate (α-TCP) and Na_₂_HPO_4_. While Na_2_HPO_4_ (Merck KGaA, Darmstadt, Germany, Lot: F2066186918) was commercially sourced, the α-TCP powder had to be produced. This involved mixing 0.716 mol of CaHPO_4_ and 0.33 mol of CaCO_3_ in agate jars using a planetary mill for one hour. The powder was transferred into crucibles, combined with a sintering ring, and sintered at 1400 °C in a high-temperature furnace. After sintering, the powder was ground to a particle size of < 355 µm, followed by four hours of dry milling. The resulting α-TCP was mixed in a 3:1 ratio with a 2.5% Na_2_HPO_4_ solution and immediately applied into the pre-fabricated silicone molds. The filled negative molds were subsequently stored for 2–4 h in a TW20 water bath (Julabo GmbH, Seelbach, Germany) at 37 °C and 100% humidity without direct water contact. The CDHA test specimens were then demolded and immersed in water at 37 °C for seven days. This was followed by two days of dehydration in a drying oven. The surfaces of the test specimens were roughened with P80 SiC wet sandpaper and cleaned using compressed air. CDHA was analyzed via X-ray diffraction before use. The samples were stored in a drying cabinet for seven days and then in a desiccator for two days. The samples were then coated with 3.5 nm platinum on the EM ACE600 sputter coater (Leica, Wetzlar, Germany) in an argon atmosphere. They were viewed on the SEM Crossbeam 340 (Zeiss, Oberkochen, Germany) at an acceleration voltage of 5 kV and a pressure of 10–5 bar and images were taken at up to 5000 × magnification.

### Biological testing: MG63 cell line studies

#### General approach

Human osteosarcoma cells (MG-63) were used as a robust osteoblast-like model for initial biocompatibility screening, offering high reproducibility despite their tumor-derived origin. To assess cell viability, proliferation, and attachment, WST-1 assay, DNA quantification (PicoGreen), and automated cell counting were performed. While WST-1 reflects mitochondrial activity as a measure of metabolic viability, DNA content and cell counts serve as indicators of total cell number. Combining these complementary methods allowed detection of potential discrepancies due to cell aggregation, detachment, or altered cell size or ploidy. This multimodal approach enabled internal validation and improved the reliability of the biocompatibility assessment.

#### Experimental setup and seeding

The MG-63 cells were obtained from ATCC and cultivated in 75 cm 3 Cellstar® cell culture flasks (Greiner Bio-One GmbH, Frickenhausen, Germany). They were incubated with just under 15 ml of culture medium at 37 °C and 5% CO_2_ in the Heracell™ 150i incubator (Thermo Fisher Scientific, Waltham, United States). The culture medium was gibco™ Dulbecco’s Midified Eagle Medium, high glucose, GlutaMAX™ (DMEM 31,966–021) (Life Technologies Limited, Paisley, United Kingdom) with the addition of 10% foetal calf serum and 1% penicillin–streptomycin. Every 4 days, 1/8 of the cells were passaged. The medium was first aspirated and the cells washed with 10 ml PBS. After adding of Accutase and incubation, the passability of the cells was checked under the X-LED 8W reflected light microscope (VWR). Now culture medium was added, which inhibited the accutase, and 1/8 of the cell suspension present was transferred to a new cell culture flask in which medium had been added. Accutase and culture medium were warmed to 37 °C before use. The remaining cells were seeded for experiments, frozen or discarded. For seeding, 100 µl of the cell suspension was pipetted into 10 ml isotonic electrolyte solution and the cell concentration was determined using the Innovatis CASY cell counter (Omni Life Science, Bremen, Germany). The cell suspension was always diluted with culture medium until the desired cell concentration was reached.

#### Assessment of cellular response—viability and proliferation

Four measurement times were defined (after two days, four days, seven days and nine days). For each test day, four sterile test specimens per cement were placed in a 24-well plate to be colonized. For each test day, a further 24-well plate was equipped with an additional test specimen per cement, which was not to be colonized and was to serve as a cement-specific sample blank for the planned measurements. In addition, cells were to be cultivated on polystyrene in four wells on this second plate as a control. To reduce the influence of potentially cytotoxic early-stage degradation products and to stabilize the pH, the cement specimens were pre-incubated in culture medium for 24 h before cell seeding. The medium was then discarded and replaced with fresh medium containing the cells. Now 50,000 MG-63 cells were seeded into each well to be colonized. This time point was considered the day 0 of the experiment. All 24-well plates were then incubated in an incubator at 37 °C and 5% CO_2_. At each measurement time, one of the plates with colonized test specimens and one with uncolonized test specimens and cells on polystyrene were removed from the incubator for the measurements. Firstly, the culture medium was removed from all wells and preserved for later ICP measurements. All test specimens were then transferred to new 24-well plates. The WST-1 assay was then performed. A medium change was also carried out on the remaining 24-well plates at the measurement times, i.e. on test days 2, 4 and 7.

The WST-1 reagent was diluted 1:10 with medium and 600 µl was pipetted into each well, including blank determination. This was followed by a 30-min incubation at 37 °C and 5% CO_2_. 200 µl were then quickly transferred twice from each well to a 96-well plate for a duplicate determination. Any air bubbles were punctured with a cannula and the absorbance was measured at a wavelength of 450 nm with a reference filter at 690 nm using the SPARK® 20M photometer (Tecan Trading AG, Männedorf, Switzerland).

For the quantification of cell proliferation, both a Casy cell count and a Picogreen assay were connected to the WST-1 assay. First, the cells were detached and counted as described above. The cell suspension was transferred from each well into a SafeSeal reaction tube and centrifuged so that a clear cell pellet was usually visible. After the supernatant liquid was removed, lysis buffer (0.1% Triton-X in 1x-PBS solution) was added and resuspended until the pellet was dissolved. After 60 min storage on ice, all cell lysates were returned to the 24-well plates so that any remaining cells could also be lysed. This was followed by three freezing and thawing steps and the PicoGreen assay for the detection and quantification of dsDNA. The Quant-IT™ PicoGreen™ dsDNA Assay Kit (Life Technologies Corporation, Eugene, United States) was used. 1 ml of the lysis buffer 0.1% Triton X-100 (Sigma Aldrich Chemie GmbH, Steinheim, Germany, Lot: SLCF5969) in single-concentration PBS solution was pipetted into each well. The 24-well plate was frozen three times at − 24 °C and thawed again. A 100 µg/ml DNA solution was diluted 1:50 with single concentrated TE buffer. The 2 µg/ml DNA solution was used to prepare DNA standards with final concentrations of 1–1000 ng/ml DNA by dilution with TE buffer. The PicoGreen working solution was prepared by diluting the PicoGreen reagent 1:200 with 1 × TE buffer, stored in the dark and used quickly. All these solutions were also cooled on ice. Twice 100 µl each of the standards and the cell lysates were pipetted into the black microplate 96-well plate (Greiner Bio-One GmbH, Kremsmünster, Austria) for duplicate determination. In addition, only TE buffer was added to two wells as a standard blank and only lysis buffer as a sample blank. Subsequently, 100 µl of the Picogreen working solution was pipetted into each well and any air bubbles any air bubbles with a cannula. Using the Tecan photometer a fluorescence measurement was carried out by measuring the emission at 535 nm under excitation with light of wavelength 485 nm. All solutions used were quickly consumed and stored on ice when not in use. The whole experiment was then repeated.

In summary, four cement discs per composition were analyzed per test day in two independent runs (n = 8 per group per time point) for cell counting, WST-1, and PicoGreen assays. In WST-1 and PicoGreen assays, two aliquots were measured per disc, resulting in a total of n = 16 measurements per group per time point.

### Biological testing: RAW 264.7 cell line studies

#### Experimental design and seeding

In addition to the MG-63-based investigations, experiments were carried out with the osteoclast cell line RAW 264.7, which was cultivated in the same culture medium. However, the cells were detached from the bottom of the cell culture flask before passing through a cell scraper. Medium was first aspirated and the cells were washed with PBS before medium was added. 250 µl of the cell suspension was transferred to a new bottle containing 15 ml of culture medium. The cells were passaged every three to four days.

Three series of measurements were carried out for the test over a period of 15 days. Three measurement times were defined: after six days, after ten days and after 15 days. One 96-well plate was loaded with test specimens for each measurement series. The first series of measurements was used to quantitatively determine the TRAP activity and DNA concentration of the cells colonizing the test specimens. Here, each of the three 96-well plates was loaded with four test specimens to be colonized per cement and one test specimen not to be colonized as a blank. In addition, cells were cultivated on polystyrene in four wells as a control. In the second and third series of measurements, cells on the cements were to be qualitatively detected using TRAP staining and scanning electron microscopy. For this purpose, two test specimens to be colonized per cement were placed in the respective plates.

After placing the sterile test specimens, they were incubated for 24 h with 200 µl of the culture medium. The medium was then removed and discarded, after which 6400 cells were seeded into each well to be colonized by adding 200 µl of medium containing 32,000 cells/ml. After another 24 h, the medium was again removed and discarded and 200 µl medium with 50 ng/ml RANKL was added. This time point was day 0 of the experiment. The medium was changed and 200 µl of culture medium with 50 ng/ml RANKL was added again on test days three, six, eight, ten, 13 and 15. Test series 1 was then repeated to verify the results.

#### Assessment of cellular response—osteoclastic differentiation and activity

To investigate the differentiation using the TRAP assay, an acetate-tartrate substrate buffer (AT buffer) was first prepared, which is a solution with 100 mM Na acetate (Sigma-Aldrich Chemie GmbH, Steinheim, Germany, Lot: SLCK6020) and 50 mM Na tartrate (Sigma Aldrich Chemie GmbH, Steinheim, Germany, Lot: BCCD8753) in double-distilled water. This was adjusted to pH 6.1 with 100 mM acetic acid. By dissolving para-nitrophenol (Sigma Aldrich Chemie GmbH, Steinheim, Germany, Lot: SHBN8994) in 1 ml of the AT buffer, a 5 mM pNP solution was obtained, which was used with AT buffer to prepare a standard series (1.95 µM to 500 µM pNP). A solution of 7.5 mM para-nitrophenyl phosphate (pNPP) (‘Phosphatase substrate’, Sigma Aldrich Chemie GmbH, Steinheim, Germany, Lot: SLCN6518; ‘4-Nitrophenylphosphat Dinatriumsalz Hexahydrat, Carl Roth GmbH, Karlsruhe, Germany, Lot: 204,349,911) in AT buffer was prepared as a substrate solution. All these solutions were always freshly prepared shortly before performing the assay.

After thawing the cell lysates to be analyzed, they were first transferred to a 1.5 ml SafeSeal reaction tube and centrifuged at 15,000 g for 5 min so that some of the cement residues settled and the cell lysates, separated from the cement residues, could be transferred to new tubes. Subsequently, 200 µl each of the standards and the standard blank (AT buffer) and 50 µl each of the samples and the sample blank (lysis buffer) were added to a transparent 96-well plate to determine the duplicate values. After adding 150 µl of substrate solution to each of the wells with cell lysates and sample blank, incubation took place for 60 min at 37 °C and 5% CO_2_ in an incubator. The reaction was then stopped by adding 50 µl of a 3 M NaOH solution to each well. If bubbles were present, these were punctured using a cannula. The absorbance at 405 nm and reference wavelength of 620 nm could then be measured on the Tecan photometer and converted into the concentration of the resulting pNP using the standard series.

In preparation for the Picogreen assay to quantify cell proliferation, a 1 × -TE buffer was first prepared by diluting a 20x-TE buffer with DNA-free ultrapure water. A 200 ng/ml DNA standard was created by diluting a 100 µg/ml λ-DNA solution in this 1x-TE buffer. A standard series (0.195 ng/ml to 200 ng/ml DNA) was created by diluting this standard with 1x-TE buffer according to a geometric series. In addition, the Picogreen reagent was diluted 800-fold in 1 × TE buffer to prepare a Picogreen working solution and stored in the dark until use. All these solutions were freshly prepared prior to the assay and cooled on ice at all times.

Following the TRAP assay, twice 20 µl of all standards and the standard blank (1x-TE buffer), as well as twice 20 µl of all cell lysates and the sample blank (lysis buffer) were added to a black 96-well plate in the sense of a double value determination. Now 180 µl of Picogreen working solution was pipetted into each well and any air bubbles that may have formed were broken. This was followed by a 5-min incubation at room temperature in the absence of light and oscillation at 100 rpm. Fluorescence was measured on a Tecam photometer under excitation with light at a wavelength of 485 nm and detection of the emission at 535 nm.

For PicoGreen assay and TRAP assay using the RAW 264.7 cell line, four cement discs per composition were analyzed per test day in two independent runs (n = 8 per group per time point). Two aliquots were measured per disc, resulting in a total of n = 16 measurements per group per time point.

The Phosphatase Acid Kit (SigmaAldrich Chemie GmbH, Steinheim, Germany) was used for the qualitative detection of TRAP. At each measurement time, a fixing solution consisting of 765 µl citrate solution, 1.990 ml acetone and 245 µl 37% formaldehyde and a coloring solution were prepared from the components of the phosphatase acid kit according to the manufacturer’s instructions. A 96-well plate assigned to measurement series 2 was then removed from the incubator and the medium was removed from the wells. Now 100 µl of fixing solution was added to each cement test specimen to be colored and removed again after 30 s. After two thorough washes of the cement surfaces in 1 × PBS solution, 100 µl of coloring solution was pipetted into each well. This was followed by a 60-min incubation at 37 °C and 5% CO_2_ in an incubator. The coloring solution was then removed and the test specimens were dried for 7 days after washing the surfaces again in double-distilled water. They were then viewed under a LEICA DMS1000 reflected light microscope at 20 × magnification. The staining was performed on four cement discs per group and time point. Representative images are shown in the result section.

#### Morphological evaluation

In preparation for scanning electron microscopy, the test specimens in the third series of measurements were washed twice with 1 × -PBS solution and fixed to the cement surfaces by overcoating for 15 min with 6% glutaraldehyde, which was prepared by diluting a 25% glutaraldehyde solution (SigmaAldrich Chemie GmbH, Steinheim, Germany, Lot: SLCM5878) with 1 × -PBS. The samples were then transferred to 24-well plates and washed five times for five minutes on ice in 1x-PBS. The test specimens were then dehydrated using an ascending ethanol series with 30%, 50%, 70%, 90% and 100% ethanol for a total of 290 min, after which they were coated twice for 15 min with hexamethyldisilazane (SigmaAldrich Chemie GmbH, Steinheim, Germany, lot: SHBG7971V). After removal of the hexamethyldisilazane, the test specimens were stored in a desiccator until further use. They were then fixed and sputtered as described above and viewed under the SEM. Representative data from one specimen per composition are shown in the result section.

### Ion release analysis (ICP-MS)

The ICP mass spectrometer iCAP™ RQ (Thermo Fisher Scientific, Waltham, United States) was used to analyse the release of ions from the test specimens into the medium. The supernatants of the culture medium were transferred to 1.5 ml reaction vessels on each test day and with each additional medium change. For each cement composition, four colonized test specimens (n = 4) were analyzed per time point in two independent batches. Parallel to cell cultivation during the experiments, four uncolonized test specimens per cement (n = 4) were placed in the same amount of medium and this medium was also preserved during each medium change. The tubes were centrifuged at 15,000 g for 5 min and the liquid supernatant was pipetted into a new tube without the cement pellet at the bottom. These samples were diluted 1:50 with 0.69% nitric acid. The measurements were carried out against the ICP standards for calcium, magnesium and phosphorus (all from Merck KGaA, Darmstadt, Germany). The culture medium served as a blank. The ion concentrations present in it were subtracted from all measured values as part of the analysis. To differentiate among total, passive, and active resorption or adsorption, we defined the following approach: “Total” refers to ion concentrations measured in the supernatants of cell-colonized specimens, reflecting both passive material degradation and cell-mediated processes. “Passive” values were obtained from uncolonized specimens incubated in parallel, representing ion release or adsorption without cellular influence. “Active” resorption or adsorption was calculated as the difference between total and passive values.

### Statistical analysis

Statistical analysis was performed using OriginPro (OriginLab Corporation, Northampton, MA, USA). One-way and two-way ANOVA were applied as appropriate, followed by Tukey’s HSD post hoc test. A significance level of α = 0.05 was applied in all tests. Only selected relevant statistically significant differences are displayed in the graphs to preserve clarity. Significance levels are indicated as follows: *p* < 0.05 (*), p < 0.01 (**), p < 0.001 (***).

## Data Availability

The datasets generated and/or analysed during the current study are not publicly available, as they are the basis of ongoing yet unpublished studies, but are available from the corresponding author on reasonable request.
